# Bleeding Risk Following Stereotactic Body Radiation Therapy for Localized Prostate Cancer in Men on Baseline Anticoagulant or Antiplatelet Therapy

**DOI:** 10.3389/fonc.2021.722852

**Published:** 2021-09-17

**Authors:** Abigail Pepin, Sarthak Shah, Monica Pernia, Siyuan Lei, Marilyn Ayoob, Malika Danner, Thomas Yung, Brian T. Collins, Simeng Suy, Nima Aghdam, Sean P. Collins

**Affiliations:** ^1^George Washington University School of Medicine and Health Sciences, Washington, DC, United States; ^2^Department of Radiation Medicine, Georgetown University Hospital, Washington, DC, United States; ^3^Department of Radiation Medicine, Harvard, Boston, MA, United States

**Keywords:** stereotactic body radiation therapy, anticoagulation, antiplatelet, bleeding risk, prostate cancer

## Abstract

**Purpose:**

Patients on anticoagulant/antiplatelet medications are at a high risk of bleeding following external beam radiation therapy for localized prostate cancer. SBRT may reduce the bleeding risk by decreasing the volume of bladder/rectum receiving high doses. This retrospective study sought to evaluate the rates of hematuria and hematochezia following SBRT in these patients.

**Methods:**

Localized prostate cancer patients treated with SBRT from 2007 to 2017 on at least one anticoagulant/antiplatelet at baseline were included. The minimum follow-up was 3 years with a median follow-up of 72 months. Patients who had a rectal spacer placed prior to SBRT were excluded. Radiotherapy was delivered in 5 fractions to a dose of 35 Gy or 36.25 Gy utilizing the CyberKnife system. Hematuria and hematochezia were prospectively assessed before and after treatment using the Expanded Prostate Cancer Index Composite (EPIC-26). Toxicities were scored using the CTCAE v4. Cystoscopy and colonoscopy findings were retrospectively reviewed.

**Results:**

Forty-four men with a median age of 72 years with a history of taking at least one anticoagulant and/or antiplatelet medication received SBRT. Warfarin (46%), clopidogrel (34%) and rivaroxaban (9%) were the most common medications. Overall, 18.2% experienced hematuria with a median time of 10.5 months post-SBRT. Altogether, 38.6% experienced hematochezia with a median time of 6 months post-SBRT. ≥ Grade 2 hematuria and hematochezia occurred in 4.6% and 2.5%, respectively. One patient required bladder neck fulguration and one patient underwent rectal cauterization for multiple non-confluent telangiectasia. There were no grade 4 or 5 toxicities. Cystoscopy revealed bladder cancer (40%) and benign prostatic bleeding (40%) as the most common hematuria etiology. Colonoscopy demonstrated hemorrhoids (54.5%) and radiation proctitis (9.1%) as the main causes of hematochezia. There was no significant change from the mean baseline EPIC-26 hematuria and hematochezia scores at any point during follow up.

**Conclusion:**

In patients with baseline anticoagulant usage, moderate dose prostate SBRT was well tolerated without rectal spacing. High grade bleeding toxicities were uncommon and resolved with time. Baseline anticoagulation usage should not be considered a contraindication to prostate SBRT.

## Introduction

Post-treatment quality of life remains an important consideration when selecting prostate cancer treatment. Post-treatment bleeding including hematochezia and hematuria are known bothersome late side effects of radiation therapy (1). The incidence of grade 2 or worse gross hematuria after conventionally fractionated external beam radiation therapy (EBRT) is estimated to be <5% (2). Some studies report post-treatment proctitis including rectal bother and bleeding to occur in 5-20% of patients after undergoing conventionally fractionated treatment (3). A number of factors can influence a patient’s individual risk of developing radiation-induced genitourinary (GU) and gastrointestinal (GI) bleeds including age, co-morbidities, history of symptomatic hemorrhoids, treatment technique and/or anticoagulation.

Anticoagulation is utilized to prevent clotting in patients with a range of cardiovascular diseases including atrial fibrillation, venous thromboembolism, ischemic heart disease and valvular disease (4). Similar to prostate cancer, these diseases are prevalent in the elderly population and the incidence is increasing. Bleeding is a common risk of anticoagulation, and radiation therapy may increase the risk (4). Risk factors for anticoagulant-induced bleeding include older age, race, obesity, comorbidities and utilization of combination therapy (4).

Prostate radiation therapy (RT) may increase this risk of clinically significant bleeding in men on anticoagulation (1, 5). Endoscopic findings associated with proctopathy or cystopathy can include telangiectasias, congested mucosa, or ulcers (6). Post-RT bleeding is secondary to chronic radiation-induced vascular ectasias which are characterized by friability and increased permeability (7). Anticoagulation, by disrupting normal hemostasis, may convert mild ectasias’ bleeding into clinically significant bleeding (8). Patients on anticoagulants had a high rate of bleeding from external beam radiation therapy when compared to patients that were not on anticoagulants (1). The absolute risk of hematuria or hematochezia was 39% (1). Hematochezia was more common than hematuria. The 4-year actuarial risk of Grade 3 or worse bleeding toxicity was 15.5% (1). In many cases, the bleeding did not fully resolve even with surgical intervention (1). Higher radiation dose was associated with an increased risk of Grade 2 or worse bleeding (1). Choe et al. identified dose volume histogram (DVH) guidelines including rectal V_50_ <50% and V_70_ <10% to be below the threshold for which Grade 3 bleeding events occurred (1).

The use of stereotactic body radiation therapy (SBRT) in the treatment of localized prostate cancer has been determined to be safe and efficacious in several ongoing multi-institutional trials (9, 10). The impact of baseline anticoagulation use during and following SBRT for prostate cancer on gastrointestinal and genitourinary bleeds remains unknown to date. In this report, we sought to report on the impact of baseline anticoagulation and/or antiplatelet usage on the risk of bleeding following SBRT.

## Methods

### Patient Selection

The Georgetown University Institutional Review Board approved this single institution review (IRB#2009-510). All individuals who underwent SBRT for treatment of their localized prostate cancer at MedStar Georgetown University Hospital from 2007 to 2017 were eligible for inclusion if they were on anticoagulation at time of initial consultation. Anticoagulants included oral anticoagulants and antiplatelet medications. Patients on low dose aspirin were excluded. Patients were required to have a minimum of three years of follow up to be included.

### SBRT Treatment Planning and Delivery

Simulation, contouring, and treatment planning were performed using our institutional protocol (11). Patients underwent a treatment planning CT and pelvic MRI at least one week after placement of 4 to 6 gold fiducial markers in the prostate. The clinical target volume (CTV) included the prostate and proximal seminal vesicles. The planning target volume (PTV) was expanded 3 mm posteriorly and 5 mm in all other directions from the CTV. The bladder and rectum were contoured structures that were evaluated on dose-volume histogram analysis during treatment planning using Multiplan (Accuray Inc, Sunnyvale, CA) inverse treatment planning. Five fractions of 7-7.25 Gy were delivered to the PTV over one to two weeks.

The bladder volume receiving 37 Gy was limited to ≤ 5 cc and the rectal volume receiving 36 Gy was limited to ≤ 1 cc. Additional bladder dose constraints included volume less than 40% receiving 50% of prescribed dose and volume less than 10% receiving less than 100% of the prescribed dose. For the rectum, secondary dose constraints included volume less than 40% receiving 50% of prescribed dose, volume less than 25% receiving 75% of prescribed dose, volume less than 20% receiving 80% of the dose, volume less than 10% receiving 90% of the dose, and volume less than 5% receiving 100% of prescription dose.

### Follow-Up and Statistical Analysis

Toxicities were assessed during follow up visits at one-month post treatment, every three months for the first year, every 6 months in the second year, then yearly and scored using the common terminology criteria for adverse events (CTCAE) v4. Acute bleeding was defined as experiencing toxicity within 6 months of radiation therapy. Late bleeding was defined as occurring at least 6 months after delivery of radiation therapy. Grade 1 represents minimal bleeding not requiring medications. Grade 2 indicates bleeding requiring new medication or minor rectal laser coagulation. Grade 3 toxicity indicates severe bleeding that required surgical intervention. Cystoscopy and colonoscopy were recommended for the initial evaluation of bleeding and were reviewed for this study. Rectal Telangiectasia were graded using the Vienna Rectoscopy Score (VRS): Grade 1 (a single telangiectasia), Grade 2 (multiple non-confluent telangiectasia) and Grade 3 (multiple confluent telangiectasia).

Cross-sectional assessment of quality of life using Expanded Prostate Cancer Index Composite (EPIC-26) questionnaires were assessed on the first day of treatment and during the follow up visits at one-month post treatment, every 3 months during the first year post-SBRT, every 6 months after the second year, and then yearly. The patient scores for EPIC-26 questions related to hematochezia and hematuria were determined using a weighted average. Minimally important differences were computed by obtaining half the standard deviation at baseline.

## Results

Forty-four patients on baseline anticoagulation were treated with SBRT for their localized prostate cancer between 2006 and 2017. The median follow-up of 72 months. Patient characteristics are listed in **Table 1**. The patients were ethnically diverse with a median age of 71.5 years (range 57-84 years). Comorbidities were common (Carlson Comorbidity Index ≥ 1 in 66%). Our cohort included a diverse variety of BMI statuses including 32% of patients who were obese (BMI > 30). One patient had a prior transurethral resection of the prostate (TURP). Warfarin (46%), clopidogrel (34%) and rivaroxaban (9%) were the most common medications. Other anticoagulant and antiplatelet agents used included enoxaparin, apixaban, dabigatran, aspirin, and Aggrenox. Two patients were on combination therapy (4.5%). The most common indication for anticoagulation was atrial fibrillation (25%). Other indications included a history of coronary artery disease (CAD), cerebrovascular accident/transient ischemic attack (CVA/TIA), deep venous thrombosis (DVT), heart valve deformity. Eighteen percent of individuals had multiple indications for anticoagulation. Per the D’Amico Risk Classification, 9 patients were low risk, 28 were intermediate risk, and 7 patients were high risk. Five patients received androgen deprivation therapy (ADT). Sixty eight percent of the patients were treated with 36.25 Gy in five fractions.

**Table 1:** Patient characteristics and treatment.

	Percent of Patients
	(n = 44)
**Age (years): Median 71.5 (57-84)**	
50-59	6.8% (3)
60-69	29.5% (13)
70-79	47.7% (21)
>80	15.9% (7)
**Race**	
White	52.3% (23)
Black	45.5% (20)
Other	2.3% (1)
**BMI**	
18.5-24.9	34.1% (15)
25.0-29.9	34.1% (15)
>30.0	31.8% (14)
**Prior urologic procedure**	
Yes	2.3% (1)
No	97.7% (43)
**Charlson Comorbidity Index**	
0	34.1% (15)
1-2	59.1% (26)
>2	6.8% (3)
**Anticoagulation/antiplatelet**	
Warfarin	45.5% (20)
Clopidogrel	34.1% (15)
Rivaroxaban	9.1% (4)
Enoxaparin	2.3% (1)
Other	4.6% (2)
Combination	4.5% (2)
**T stage**	
T1c-T2a	81.8% (36)
T2b-T2c	18.2% (8)
**Gleason Score**	
6	31.8% (14)
7	59.1% (26)
8-9	9.1% (4)
**Risk group (D’Amico)**	
Low	20.5% (9)
Intermediate	63.6% (28)
High	15.9% (7)
**Hormone Therapy**	
Yes	11.4%
No	88.6%
**SBRT dose**	
35	31.8% (14)
36.25	68.2% (30)

Patients experienced both acute and late bleeding events (**Table 2**). In the acute setting, 22.7% of patients experienced an acute Grade 1 bleed, of which the majority (80%) were secondary to rectal bleeding. There were no Grade 2 bleeding events. One individual experienced an acute Grade 3 bleed. This patient experienced hematochezia at 6 months requiring cauterization. In the late setting, 27.3% of patients experienced late Grade 1 bleeding events. One individual experienced a late grade 2 hematuria event, and one individual experienced a late grade 3 hematuria event requiring fulguration.

**Table 2:** Cumulative incidence of acute and late CTC-graded hematuria and hematochezia.

	None	Grade 1	Grade 2	Grade 3
**ACUTE**				
Hematuria	42	2	0	0
Hematochezia	35	8	0	1
Overall	33 (75.0%)	10 (22.7%)	0 (0%)	1 (2.3%)
**LATE**				
Hematuria	38	4	1	1
Hematochezia	33	11	0	0
Overall	30 (68.2%)	12 (27.3%)	1 (2.3%)	1 (2.3%)

Six patients had cystoscopies. The findings can be found in **Table 3**. Two individuals were found to have bladder cancer. One individual was found to have a bleeding local recurrence. Two individuals were found to have benign prostatic bleeding. The remaining individual was found to have normal cystoscopies. Twenty-three individuals underwent colonoscopy in the months to years following treatment (**Table 4**). The most common finding were hemorrhoids. Three individuals were found to have radiation proctitis with multiple non-confluent telangiectasia (VRS Grade 2).

**Table 3:** Results of cystoscopies.

Patient	Age	CCI	Anticoagulant Use	Time to Cystoscopy	Cystoscopy Findings
1	83	1	Plavix	1 year	Bladder Cancer
2	63	2	Warfarin	6 years	Prostatic Recurrence
3	62	2	Xarelto	4 years	Benign Prostatic Bleeding
4	71	1	Plavix	5 years	Benign Prostatic Bleeding
5	66	1	Plavix	9 years	Bladder cancer

**Table 4:** Results of colonoscopies.

Patient	Age	CCI	Anticoagulant Use	Time to Colonoscopy	Colonoscopy Findings
1	74	1	Warfarin	1 year, 3 years	Hemorrhoids
2	74	0	Warfarin	1 year 6 mon	Radiation proctitis (VRS Grade 2)
3	67	1	Plavix, ASA	8 years	Hemorrhoids
4	75	0	Warfarin	2 years 6 mon	Radiation proctitis (VRS Grade 2)
5	63	0	Warfarin	2 years	Hemorrhoids
6	63	2	Warfarin	3 years, 6 years	Hemorrhoids
7	63	1	Warfarin	6 mon	Hemorrhoids
8	80	1	Plavix	1 year 6 mon	Hemorrhoids
9	59	4	Plavix	9 mon	Radiation proctitis (VRS Grade 2)
10	67	2	Plavix	1 year, 4 years	Hemorrhoids
11	71	3	Warfarin	3 years	Hemorrhoids
12	58	2	Warfarin	1 year 6 mon	Hemorrhoids
13	66	1	Plavix	3 years	Hemorrhoids
14	72	2	Plavix	4 years	Hemorrhoids

**Figure 1:**
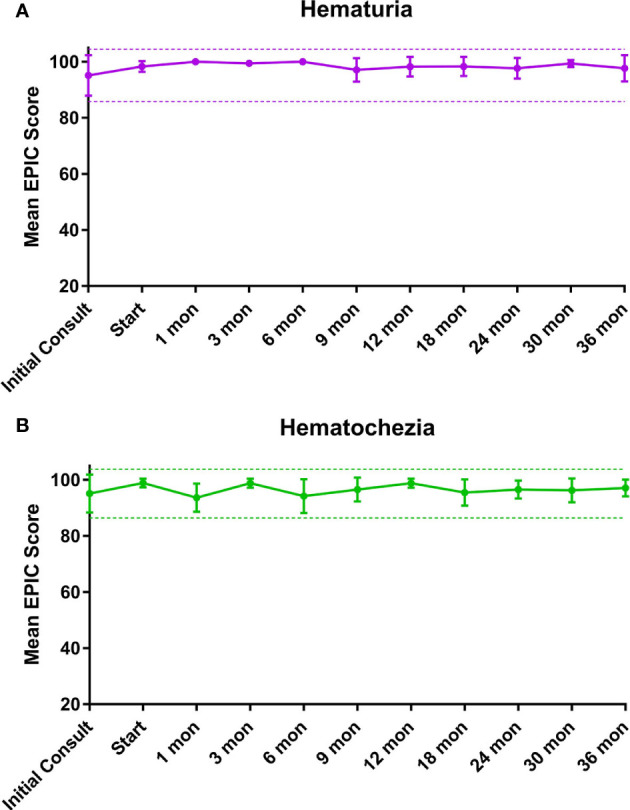
Mean summary scores at baseline and following SBRT for prostate cancer. **(A)** Hematuria. **(B)** Hematochezia. Thresholds for clinically significant changes in scores (½ standard deviation above and below the baseline) are marked with dashed lines. EPIC scores range from 0–100 with higher values representing a more favorable health-related QOL.

EPIC-26 hematuria and hematochezia scores following SBRT can be found in **Figures 1A, B**, respectively. Overall, 18.2% experienced hematuria with a median time of 10.5 months post-SBRT (**Table 5**). At the time of the initial consultation, 3.7% of our cohort reported bothersome hematuria (**Table 5**). Hematuria bother increased following treatment and peaked at 9 months post treatment with 2.3% of patients reporting that it was a moderate to big problem from 9-24 months post-SBRT (**Table 4**). Hematuria bother returned to baseline by 30 months after SBRT. At 36 months, 2.3% reported hematuria as being a very small to small problem with no patients reporting hematuria as being a moderate to big problem. There were no clinically significant changes in hematuria at any time point following treatment (**Figure 1A**: MID 3.2).

**Table 5:** Bleeding following SBRT for prostate cancer: hematuria (patient-reported responses to Question 4c of the EPIC-26) and hematochezia (patient-reported responses to Question 6d of the EPIC-26).

	Initial Consult	Start	1 mon	3 mon	6 mon	9 mon	12 mon	18 mon	24 mon	30 mon	36 mon
**Hematuria **											
No problem	96.3%	93.2%	100.0%	97.7%	100.0%	95.3%	97.7%	97.7%	95.3%	97.5%	97.7%
Very Small- Small problem	3.7%	6.8%	0.0%	2.3%	0.0%	2.3%	0.0%	0.0%	2.3%	2.5%	2.3%
Moderate - Big problem	0.0%	0.0%	0.0%	0.0%	0.0%	2.3%	2.3%	2.3%	2.3%	0.0%	0.0%
**Hematochezia**										
No problem	92.6%	95.5%	83.7%	95.3%	88.4%	93.0%	95.3%	90.9%	88.4%	90.0%	90.7%
Very small- Small problem	7.4%	4.5%	14.0%	4.7%	7.0%	4.7%	4.7%	6.8%	11.6%	7.5%	9.3%
Moderate - Big problem	0.0%	0.0%	2.3%	0.0%	4.7%	2.3%	0.0%	2.3%	0.0%	2.5%	0.0%

Altogether, 38.6% experienced hematochezia with a median time of 6 months post-SBRT. At the time of the initial consultation, 7.4% of patients reported bothersome hematochezia; however, no patient felt it was a moderate to big problem (**Table 5**). At 1 month post-SBRT, this increased to 14% reporting rectal bleeding as being a very small to small problem and 2.3% reporting the bleeding to be a moderate to big problem. A few patients experienced transient episodes of bothersome rectal bleeding over the next three years. At 36 months, 90.7% reported having no problems with hematochezia. Nine percent of patients reported hematochezia; however, no patient felt it was a moderate to big problem. There were no clinically significant changes in the months following treatment with respect to hematochezia (**Figure 1B**; MID 2.6).

## Discussion

Chronic anticoagulation therapy alone may increase an individual’s risk of developing hematuria and or hematochezia (12). The yearly incidence of major bleeding is 2-5% (13). As seen in this manuscript, occult malignancies, benign prostatic bleeding, and/or benign acute lower gastrointestinal bleeding such as hemorrhoids where common sources of non-radiation related bleeding in our patients on anticoagulants (14–16). Benign bleeding from enlarged prostates and diverticular disease is are common causes of bleeding in the aging population. Like irradiated tissue, tumor vasculature is friable and prone to bleeding (17). The risk of bleeding is highest in urinary and colorectal cancers (14, 18).

The risk of radiation induced hematuria is dependent upon the total radiation dose and the volume of the bladder in the high dose region (19). Our group has previously reported on the incidence of hematuria in unselected patients who had undergone SBRT for their localized prostate cancer (20). Similar to the present study, 18.3% experienced at least one episode of hematuria following SBRT, and the 3-year actuarial incidence of late ≥ grade 2 hematuria was 2.4% (20). On multivariate analysis, history of prior benign prostatic hyperplasia (BPH) procedure(s) (p = 0.002) was significantly associated with the development of hematuria. Unexpectantly, it did not find an association between anticoagulation use and hematuria, despite previous reports of an association (1, 20). We hypothesize that the low rate of significant hematuria in the current study was at least partially due to the low incidence of prior urologic procedure for BPH (2.3%) in this patient population.

Our group has also previously reported on the incidence of post-SBRT rectal bleeding in unselected patients (21). In that study, 22.7% of patients reported rectal bleeding post-SBRT. In the current report, 38.6% of patients on baseline anticoagulants experienced rectal bleeding post-SBRT. Twenty five percent of patients experienced late Grade 1 hematochezia, higher than was previously reported. There were no late grade 2 or 3 rectal bleeding events. Patient’s experienced peak of hematochezia representing a problem at 1 month following treatment. This is consistent with hematochezia secondary to increased bowel frequency seen acutely following treatment. The remainder of the peaks in burden appear to be episodic in nature likely due to hemorrhoidal bleeding. By 36 months, no individuals reported hematochezia to be a moderate or big problem. These results are consistent with our previously reported findings (20, 21). In our patient population, 23 individuals underwent colonoscopies in months to years after their treatment for localized prostate cancer. No occult malignancies were detected, though polyps were noted in 60.8% of colonoscopies. The most common finding was hemorrhoids. Presence of hemorrhoids has been reported to be a strong predictor for hematochezia previously (22). Our previous report on endoscopic findings reported a rate of telangiectasias in 20% of post-SBRT patients compared to 32-88% in patients who had undergone 3D-conformal radiation therapy (3D-CRT) or intensity modulated radiation therapy (IMRT) (6). In the present study, three patients (13%) were noted to have radiation proctitis with Vienna Score 2 telangiectasias; two of the three individuals experienced symptomatic rectal bleeding. Given that bleeding was most commonly secondary to hemorrhoidal bleeding, in the authors’ opinion, anticoagulation should not be an indication for rectal spacing in patients treated with moderate dose robotic SBRT.

Dosimetric parameters may influence rates of GU and GI bleed. Total radiation dose and volumes of urethra and bladder neck exposed impact the risk of developing radiation-induced hematuria, but specific dosimetric constraints to limit late hematuria have been difficult to identify (19). The low level of high-grade hematuria in this study was likely secondary to the small number of patients with prior transurethral resection of the prostate (TURP) which qualified (20). Musunuru et al. looked at predictive factors for developing symptomatic hematochezia in patients with prostate cancer following 5-fraction linac-based SBRT (22). In that trial, Grade 2 and ≥Grade 3 late hematochezia was observed in 19.4% and 3.1% of their cohort, respectively (22). Analysis of receiver operating characteristic (ROC) curves revealed that the volume of rectum receiving 38 Gy (V38) was the strongest predictor of Grade 2 late hematochezia (22). Approximately 9% of patients who received a rectal V38 <2 cc had symptomatic rectal bleeding compared to 28% of patients who received V38 ≥2 cc (22). However, that paper used a posterior PTV margin of 4-5 mm, while our institution favors rectal sparing using a posterior PTV margin of 3 mm, which can be achievable using motion tracking (22). In this study, no patient received 36 Gy to greater than 1 cc of the rectum providing a rationale for our low rate of symptomatic rectal telangiectasia.

Our study has several limitations. It is inherently limited by its retrospective nature. Our patients were all on documented anticoagulation at time of initial consult. However, it is unknown in our study if patients were removed from anticoagulation in the weeks to years following radiation therapy. A study evaluating the risk of rectal bleeding based on timing of anticoagulation during or after radiation therapy found that anticoagulation during treatment was associated with an increased risk of bleeding, though initiation of anticoagulation after completion of radiation therapy did not significantly increase the risk of rectal bleed (23). In addition, given that we did not perform regular urinalysis on patients, the true incidence of microscopic hematuria may be higher than reported. We did not perform routine baseline cystoscopy or colonoscopy screening. As such, baseline causes of hematuria or hematochezia could not be assessed. However, patients were treated on average one month after gold marker placement, and it is possible bleeding events could have lingered from that procedure.

## Conclusion

In patients with baseline anticoagulant usage, moderate dose prostate SBRT was well tolerated without rectal spacing. High grade bleeding toxicities were uncommon and resolved with time. Baseline anticoagulation usage should not be considered a contraindication to prostate SBRT.

## Author’s Note

Portions of this research were presented in abstract form at ESTRO 2021.

## Data Availability Statement

The datasets presented in this article are not readily available because the datasets presented in this article are not readily available due to patient privacy concerns. Requests to access the datasets should be directed to the corresponding author. Requests to access the datasets should be directed to Sean.P.Collins@gunet.georgetown.edu.

## Ethics Statement

The studies involving human participants were reviewed and approved by Georgetown University IRB 2009-510. The patients/participants provided their written informed consent to participate in this study.

## Author Contributions

AP was the lead author, who participated in data collection, data analysis, manuscript drafting, table/figure creation, and manuscript revision. SSh aided in contributed to data collection. MP aided in review and revision of the manuscript. SL developed the SBRT treatment plans and contributed to data analysis. MD contributed to study design and clinical data collection. MA, TY, BC, and NA aided in review of the manuscript. SSu is a senior author who organized the data and participated in its analysis. SC was the principal investigator who initially developed the concept of the study and the design, aided in data collected, and drafted and revised the manuscript. All authors contributed to the article and approved the submitted version.

## Funding

The Department of Radiation Medicine at Georgetown University Hospital receives a grant from Accuray to support a research coordinator. We gratefully acknowledge the grant R01MD012767 from the National Institute on Minority Health and Health Disparities (NIMHD), NIH to SC. This work was supported by The James and Theodore Pedas Family Foundation.

## Conflict of Interest

SC and BC serve as clinical consultants to Accuray Inc.

The remaining authors declare that the research was conducted in the absence of any commercial or financial relationships that could be construed as a potential conflict of interest. 

## Publisher’s Note

All claims expressed in this article are solely those of the authors and do not necessarily represent those of their affiliated organizations, or those of the publisher, the editors and the reviewers. Any product that may be evaluated in this article, or claim that may be made by its manufacturer, is not guaranteed or endorsed by the publisher.
